# COVID-19 data are messy: analytic methods for rigorous impact analyses with imperfect data

**DOI:** 10.1186/s12992-021-00795-0

**Published:** 2022-01-06

**Authors:** Michael A. Stoto, Abbey Woolverton, John Kraemer, Pepita Barlow, Michael Clarke

**Affiliations:** 1 Georgetown University and Harvard T.H. Chan School of Public Health, Boston, USA; 2 Georgetown University, D.C. Washington, USA; 3 London School of Economics and Political Science, London, UK; 4 Western University, London, Canada

**Keywords:** COVID-19, Non-pharmaceutical interventions, Surveillance data, Surveillance biases, Impact analysis, Observational studies, Study design, Interrupted time-series analysis

## Abstract

**Background:**

The COVID-19 pandemic has led to an avalanche of scientific studies, drawing on many different types of data. However, studies addressing the effectiveness of government actions against COVID-19, especially non-pharmaceutical interventions, often exhibit data problems that threaten the validity of their results. This review is thus intended to help epidemiologists and other researchers identify a set of data issues that, in our view, must be addressed in order for their work to be credible. We further intend to help journal editors and peer reviewers when evaluating studies, to apprise policy-makers, journalists, and other research consumers about the strengths and weaknesses of published studies, and to inform the wider debate about the scientific quality of COVID-19 research.

**Results:**

To this end, we describe common challenges in the collection, reporting, and use of epidemiologic, policy, and other data, including completeness and representativeness of outcomes data; their comparability over time and among jurisdictions; the adequacy of policy variables and data on intermediate outcomes such as mobility and mask use; and a mismatch between level of intervention and outcome variables. We urge researchers to think critically about potential problems with the COVID-19 data sources over the specific time periods and particular locations they have chosen to analyze, and to choose not only appropriate study designs but also to conduct appropriate checks and sensitivity analyses to investigate the impact(s) of potential threats on study findings.

**Conclusions:**

In an effort to encourage high quality research, we provide recommendations on how to address the issues we identify. Our first recommendation is for researchers to choose an appropriate design (and the data it requires). This review describes considerations and issues in order to identify the strongest analytical designs and demonstrates how interrupted time-series and comparative longitudinal studies can be particularly useful. Furthermore, we recommend that researchers conduct checks or sensitivity analyses of the results to data source and design choices, which we illustrate. Regardless of the approaches taken, researchers should be explicit about the kind of data problems or other biases that the design choice and sensitivity analyses are addressing.

## Background

The COVID-19 pandemic has led to an avalanche of scientific studies. In 2020 alone, scientists are estimated to have published 100,000-200,000 papers related to COVID-19 [1]. These studies examine the virology of the pathogen, its epidemiological characteristics (such as the risk of transmission), and its clinical manifestations. Other studies examine the efficacy of medical treatments, the safety and efficacy of vaccines, and the effectiveness of “non-pharmaceutical interventions” (NPIs) to control the spread of the virus. Still others have used qualitative methods to examine the governance structures and political factors that have shaped policy responses in different jurisdictions, and to explore public and health-workers’ attitudes to and experiences of the pandemic and, more recently, vaccines.

Quantitative studies draw on many different types of data, typically aggregated at the population-level. Some studies are randomized, but most are observational. While the effort that has and continues to go into these studies is appreciated, it also must be noted that the quality of the data and methods, and hence the validity and trustworthiness of the results, is not uniformly high. Often the media are unable to separate the wheat from the chaff, making the collective effort to understand “what works” in the fight against COVID-19 even harder.

As researchers and in our role as journal editors, we are particularly aware of studies addressing the effectiveness of government actions, especially NPIs, against COVID-19. Often such studies compare COVID-19 infections, transmissions, and/or mortality in countries, regions, and/or states with differing policy responses.

Two sets of issues can undermine the ability to make valid inferences from these studies [2]. One is the well-known limitation of observational studies to distinguish factors that influence policy responses from the effects of the policies themselves [3]. To this end, Haber and colleagues have not only published on weaknesses in studies of NPIs on COVID-19 transmission during the first wave of the pandemic [4], but researchers in this group have also prepared a guide to the strengths and limitations of this sort of evidence [5]. The second category of issues, the quality of the data and indicators on which studies of NPI effectiveness are based, has received relatively less attention. Many studies, for instance, draw on COVID-19 case data compiled by Johns Hopkins University [6] or similar sources. The service these organizations provide in aggregating these data from national and subnational public health agencies and their extensive efforts to curate the data are invaluable. The curators’ best efforts, however, cannot overcome incomplete case reporting at the source. Nor can the aggregators control for differences over time and among jurisdictions in the completeness of reporting, the availability of diagnostic tests, or definitions of cases and indicators.

The aim of this article, therefore, is to describe what data are commonly used, as well as challenges with their collection, reporting and use, in order to raise awareness about the problems they cause for research studies. We also intend this article to stimulate debate and wider discussion about common pitfalls in COVID-19 evaluation studies that should be addressed.

This article is thus intended to help epidemiologists and other researchers identify a set of data issues that, in our view, must be acknowledged in order for their work to be credible. In an effort to encourage high quality research, we also provide recommendations on how to address the issues we identify. We further intend to help journal editors and peer reviewers when evaluating studies, to apprise policy-makers, journalists, and other research consumers about the strengths and weaknesses of published studies, and to inform the wider debate about the scientific quality of COVID-19 research [7].

To address these aims, we draw on our experience as editors, reviewers, and authors of studies focused on assessing the impact of public health interventions for COVID-19 and other diseases. We further review study design considerations and issues in order to identify the analytical designs that are less sensitive to the most prevalent data problems. Finally, we demonstrate how additional checks and sensitivity analyses can help identify whether results may or may not be sensitive to data issues.

## Main text

### COVID-19 data that are commonly used and issues with their collection

COVID-19 is an ongoing global phenomenon and, from the start, far more data have been available on COVID-19, in greater detail and in real time, than in any previous pandemic or disease outbreak. These data and related metrics are commonly used to inform governmental decisions about implementing NPIs, to inform public health policy, and to direct individuals’ own behavior. Yet, the volume, variety, and velocity of these data (to use “big data” terminology) typically does not allow for the quality checks that are required for rigorous research.

Both compilers and users of these data have written about the challenges of curating meaningful data and of using the resulting metrics to guide control policies [8]. Researchers drawing on these data to identify the impact of NPIs and other interventions face similar challenges. In particular, observational studies fundamentally rely on relating changes in COVID-19 cases or other outcomes to interventions at the same or earlier times, or comparing differences in COVID-19 outcomes across countries or subnational areas to the timing of interventions in the same jurisdictions. The validity of observational studies is undermined when COVID-19 trends or cross-national differences reflect differences in definitions, reporting processes, and data collection and management practices, rather than differences in the actual number of infections.

COVID-19 case and death counts are generated through the collaboration of local, regional, national and global public health agencies. As is typically true with a new pathogen, case definitions, rules for classifying a death as COVID-19 related, and the processes used to count, report, and record these events vary across jurisdictions and change over time. The proportion of infected individuals who develop symptoms, get tested, and are reported to health departments is never 100%, and is often much less. But because of variations in “testing behavior” (i.e. individuals’ awareness of the problem and their views regarding being tested, test availability, the limitations of contact tracing operations, and so on), the proportion of COVID-19 infections that are reported is not only well less than 100% but also varies across jurisdiction and time [9].

A number of organizations aggregate and curate these data: The Johns Hopkins Coronavirus Resource Center [6], Our World in Data at the University of Oxford [10], *The New York Times* [11], and *The Economist* [12], to name just a few of the more commonly used sources. Cumulative and incremental COVID-19 cases and deaths are available in tables, graphics, and maps. Rather than individual-level records, these data are typically processed into metrics, for example, the 7-day average increase in cases or per capita deaths. Data aggregators also include data on tests performed, the test positivity rate (the percentage of tests that had a positive result), the number of vaccines delivered, and so on. How the metrics are defined, however, varies. Rates of increase might be presented over 7 or 14 days, or through a sophisticated calculation of R_t_, the current effective reproductive number. The test positivity rate might include large-scale asymptomatic screening tests at universities, or only tests done for diagnostic purposes.

The European Centre for Disease Prevention and Control (ECDC) [13] and the U.S. Centers for Disease Control and Prevention (CDC) [14] aggregate data from their own constituencies as well as other global data. Due to previous efforts to harmonize data systems and working relationships with the European Union Member States and U.S. states respectively these organizations often have more comparable data than other sources, but even so comparability has been an issue with COVID-19, as is typically the case for a new pathogen.

After pulling COVID-19 data from one or a few of these sources, researchers employ these data in many different ways in their analyses: daily counts of cases or deaths, 7-day averages of these counts, cumulative numbers of cases or deaths (sometimes as of a particular date), divided (or not) by the size of the population at risk, excess mortality, changes in life expectancy, and so on. Other researchers look at growth rates of cases or estimates of R_t_, calculated in various ways. Some studies use the date of the first recorded case, or the 100th case, as the “beginning” of the pandemic in a country (or conversely, focus on countries not reporting a case by a certain date [15]). Others focus on hospitalized cases, or hospital capacity. While some of these outcomes might be more appropriate than others depending on the design of the study, the sensitivity of these outcomes to data inconsistencies should always be considered. For instance, outcomes based on *when the first case was reported to WHO would seem to be particularly sensitive to differences in the surveillance system capacities of countries.*

Studies about the impact of infection control policies require indicators of what measures were implemented, in which settings, with what intensity, on which population and when. Reflecting the differences in policy measures under study as well as the sources of data to compile them, COVID-19 policy studies can vary dramatically in the quality of the data used to describe NPIs.

Some studies use data compiled by the Blavatnik School of Government of the University of Oxford’s Covid-19 Government Response Tracker [16]. This database tracks policy measures that governments have taken to tackle COVID-19 since 1 January 2020, including school closures, travel restrictions, vaccination policies. The database covers more than 180 countries and includes scales and indices to reflect the extent of government action in different areas. One important strength of these data is the effort that goes into ensuring that the measures are recorded and coded consistently over time and among countries.

Standard databases such as the Oxford COVID-19 Government Response Tracker, however, may not contain the specific policy variables researchers want to investigate. For example, data on the timing and intensity of interventions at subnational levels might be important, but are not included. Consequently, some researchers have developed their own policy indicators based on governmental websites and other sources. However, while the measures might be more relevant to a particular research study, similar concepts may be defined differently in each curated dataset, possibly leading to confusion in comparing the results among studies. And when each team develops its own indicators based on web searches, data quality may be an issue, especially for countries outside of the researchers’ experience and language skills.

Finally, rather than study the impact of policies *per se*, some studies have investigated the intermediate impact of the interventions on behavior. Studies have explored, for instance, the impact of mobility restrictions using country-specific mobility change data obtained from the Google Global Mobility Data Source [17] and changes in adherence to NPIs associated with pandemic fatigue using self-reported data from 16 waves of the special-purpose Coronavirus Tracking Survey [18].

COVID-19 data are not ‘one size fits all.’ Given the widespread availability of COVID-19 datasets, differences in chosen metrics among researchers and studies, and the unique challenges of epidemiological and policy data, researchers must be deliberate in choosing and using their data, while reviewers must be critical in evaluating data choices made by researchers.

### Study design considerations

Many studies examining COVID-19 NPIs necessarily seek to identify “what works” to contain COVID-19 infections, transmission, morbidity, and/or mortality. However, rather than randomized trials, which are typically preferred but seldom feasible, these “impact evaluation” [2, 19, 20] studies often utilize ‘natural’ or ‘quasi-experimental’ or other causal inference methods. In the context of COVID-19 NPIs, researchers using these ‘natural’ or ‘quasi-experimental’ approaches often identify conditions in which some countries, regions, states or individuals are subjected to a particular policy and others are not, and then conduct covariate adjusted comparisons of outcomes in these groups.

In order for observational studies to draw valid causal conclusions, though, they must use suitable designs. Haber and colleagues have identified a number of important considerations for causal impact evaluations for COVID-19 [4]. They have also identified some designs that are more effective than others, especially regarding the selection of treatment and comparison units and the inability to rule out the influence of coinciding policy changes—the result of which is a guide to the strengths and limitations of this sort of evidence [5]. Additionally, and more generally, Wagenaar and Komro have described methods for evaluating NPIs that turn on legal interventions [21], and the World Bank’s ‘Impact Evaluation in Practice’ [22] also provides an accessible guide. While it is outside the scope of this paper to detail all of the many designs used in COVID-19 studies, a few that are less sensitive to common data problems are detailed below, along with some considerations for researchers to keep in mind.

One design frequently used to examine the impact of NPIs is interrupted time-series (ITS) analysis. ITS analyses examine the level and time-trend of the outcome (e.g. COVID-19 mortality) before and after an intervention (e.g. physical distancing messaging). The effect of the NPI is calculated by comparing the post-intervention outcome and trend with the predicted outcome and trend based on the trend before the intervention was introduced.

Interrupted time-series analyses focus on changes over time periods where data quality would not normally be expected to change enormously. There are nevertheless several data- and epidemiologically-related challenges in interrupted-time series studies that authors must be aware of and address in their analyses. One challenge arises from reporting lags and temporal fluctuations. Researchers may identify a change in the outcome (e.g. COVID mortality) after the intervention, inferring an effect of the NPI. However, reporting lags can mean that this ‘effect’ is instead attributable to a change in mortality from a period before the intervention was introduced. There can also be daily, weekly, or monthly fluctuations in testing behaviour or reporting – for example where COVID-19 mortality is reported on a particular day of the week but not on others, or where test-seeking varies from day-to-day (e.g. before weekends when individuals wish to participate in social gatherings). As a result, researchers may mistakenly infer an NPI has an effect whereas changes in the outcome could be due to these fluctuations.

Interrupted time-series designs are strongest when there are many time-points of data before the NPI was introduced. This enables researchers to examine typical fluctuations and address them in the modelling strategy, reducing the influence of reporting lags, regression to the mean, and seasonal fluctuations. It is also useful to have information on the factors that influence trends in the data. For example, case counts (and thus the positivity rate) reflect individuals’ perceptions of COVID-19 risks, and hence their interest in being tested. Researchers could use mortality data that is presumably less biased to obtain adjusted estimates. Finally, context specific knowledge and qualitative documentation can help researchers discern whether the assumption that the pre-existing trend would have continued to the post-intervention period is likely to be true, or if other underlying drivers of this trend may have also changed.

Analyses that combine information across “jurisdictions” (regions with uniform public health policy and regulations, from national to municipal levels of government) with temporal changes over time within these units are usually more robust to confounding by surveillance capability or test-seeking behavior and can address many limitations of both cross-sectional and interrupted time-series studies. The strongest of these designs compares temporal changes in the outcome across sub-units of a jurisdiction (e.g. states within a country) that have identical or similar surveillance and data collection practices, but differ in their exposure to NPIs at a particular time point, rather than comparing larger jurisdictions (e.g. countries). By leveraging variation both within jurisdictions and over time, these studies can control for unobserved, time-invariant differences in surveillance capabilities across units, as well as unobserved temporal changes in test-behavior and surveillance that are constant across the jurisdiction [23–27].

Comparing within-jurisdiction changes over time across sub-units is most informative when surveillance approaches and testing behaviour are known to be reasonably uniform within the jurisdiction at large and can also be expected to change uniformly over time in that jurisdiction. For example, comparing units within nations over time partially guards against confounding by spontaneous behavior change due to perceptions of overall national risk (though spontaneous behavior change may still confound when interventions are implemented in response to worsened local epidemiological situations). These designs are also stronger when they examine changes over short time periods post-NPI – during which surveillance capabilities often would be expected to be stable, and when there is a larger number of time points of data in which those ultimately exposed to the NPI were unexposed.

### Strategies to identify and address data issues

When studies are carefully designed and are guided by theory, additional checks and sensitivity analyses can help researchers determine whether the outcomes of their analyses are sensitive to plausible issues in the data. While these supplemental analyses are dependent upon choices such as research design, data sources, and model parameters, and will therefore necessarily differ study-by-study, they can broadly be thought of as investigating potential weak points within an analysis, enhancing the analysis’s credibility in the face of uncertainty.

As an initial check, researchers might consider using falsification/placebo outcomes to diagnose factors that might bias results but that are difficult to directly measure by looking at results (e.g. unaddressed bias arising from differences in surveillance capabilities) [28]. This type of approach operates by *modus tollens* logic to justify a claim that a potential biasing factor or confounder is not present, by examining whether its consequence is present. This is particularly important in cross-sectional studies, for which causal inference depends on careful accounting for variation in data systems (which is often hard to measure) between jurisdictions; although, this approach can be used with any type of design. To illustrate, a researcher interested in the effect of mask mandates on COVID-19 incidence but concerned that jurisdictions with more aggressive public health measures might also have better testing and reporting systems might use test turnaround time (lower with stronger testing systems) or the proportion of diagnosed cases that are hospitalized or severely ill (higher with weaker testing systems) to detect consequences of stronger and weaker testing systems.

Another strategy is to provide evidence on intermediate outcomes in the causal chain. For example, stay-at-home orders should drive reductions in mobility or increases in time spent at home. If evidence demonstrates that a stay-at-home order indeed drives changes in these outcomes that are synchronous with the primary outcome (e.g. COVID transmission), then researchers can be more confident that the study findings are indeed attributable to the stay-at-home order rather than surveillance capabilities or test behaviour. Alternatively, where evidence on attributed mechanisms, such as mobility, is weak, authors should undertake further tests to examine the possible influence of surveillance capability, testing behaviour and other data issues on their study findings.

Researchers also can undertake detailed examination of surveillance capabilities and other data issues in the specific units analyzed in their analysis. For example, authors can use desk research to monitor any changes in testing or reporting procedures in each unit under study. Better still, authors should use this desk research to improve the selection of units in their study by ensuring only those with similar testing and reporting procedures, and similar temporal changes therein, are included and compared. Contrary to the common push for larger datasets, this is most rigorous when researchers include fewer jurisdictions. In such circumstances, it is feasible for researchers to carefully examine the quality of the data in each unit and differences in testing procedures and behaviour, whereas examining these across a large number of units can be unwieldy.

Reflecting good research practices in epidemiologic modeling, sensitivity analyses in COVID-19 studies often include assessments of particular estimates included in the model (e.g. the basic reproduction number R_0_); comparisons of alternate lag periods (e.g. changing the time between when a policy is introduced and when that policy is expected to have an impact on the data); and leave-one-out analyses (e.g. evaluations of model results if a jurisdiction or group of jurisdictions were removed from the analysis). Less frequent, however, are sensitivity analyses that focus on potential issues inherent to the data itself, which is the focus of this analysis.

Studies relying solely upon COVID-19 case data, for example, are dependent on shifting testing rates, where the percentage of COVID-19 cases captured by the surveillance system changes over time or across locations (breaking a common but incorrect assumption of steady ascertainment rates). The reasons for such testing fluctuations are many and include issues such as supply constraints, lengthened turnaround times in periods of high demand, testing requirements to attend school, and surges around holidays as people travel to visit family. There is also an issue of contact tracing thresholds beyond which the testing of possible contacts of a case is no longer possible.

Similar data issues also exist in other COVID-19 data sources. For example, hospitalization data may not capture cases equally across various geographical areas if there are large differences in catchment areas or considerable discrepancies in healthcare seeking behaviors. Additionally, the proportion of cases hospitalized might decline as hospitals become overwhelmed with patients and are only able to accept the most severe cases at particular points in the pandemic. COVID-19 mortality data, meanwhile, may suffer from challenges in determining what constitutes a COVID-19 attributed death and differences in the amount of time it takes to report deaths, if they are reported at all. While excess mortality data must grapple with other disruptions to the norm outside of COVID-19 deaths, such as the potential impact of people delaying or refusing treatment, cancellation of elective surgeries, possible increases in mental health-related deaths, and likely decreased spreading of other infectious diseases as a result of COVID-19 interventions.

Each of these data source challenges, and any others identified by researchers, could be explored with a sensitivity analysis or by a supplemental figure showing that a particular bias is not of concern. For instance, in a study estimating COVID-19 growth rates, Hsiang et al. recognized that greater testing or higher ascertainment rates could be driving their results, and they conducted a sensitivity analysis to estimate country-level trends in case detection over their chosen study period using data from the Centre for Mathematical Modelling on Infectious Diseases [29]. Moreover, to balance the strengths and weaknesses of various COVID-19 data sources, researchers could triangulate multiple data sources in their analyses and/or they could conduct a sensitivity analysis to exchange data sources, incorporating the respective deficiencies associated with each type of data in either case. An example of this is found in Brauner et al., where their main analysis included both case and death data and a sensitivity analysis examined results when only cases or only deaths were used [30].

As noted above, the specific sensitivity analyses and additional checks conducted will depend on the chosen data and metrics, study design, and any potential data problems identified. It is incumbent upon the researcher to understand the nuances and intricacies of any datasets and of journal editors to ensure that sufficient checks and sensitivity analyses are conducted.

## Conclusions

It is commendable that many researchers are making the effort to learn from experience with COVID-19 about the impact of NPIs and other issues. These analyses have the potential to not only inform public health practice and policy during the current pandemic, but to also generate important lessons for the future. The quality of the data used to conduct these analyses, however, is sometimes not sufficient to support them. There can be problems with the completeness and representativeness of COVID-19 outcomes data as well as their comparability over time and among jurisdictions, the adequacy of policy variables and data on intermediate outcomes such as mobility and mask use, and a mismatch between level of intervention and outcome variables. Consequently, the validity of some of these results are questionable. In addition, a large number of poor-quality studies makes it difficult to synthesize results and undermines the credibility of all results in the eyes of decision-makers and the public.

The ideal strategy to address these problems, of course, is to identify better quality data. Some analysts have concluded, for instance, that excess mortality estimates can be reliable, especially for international comparisons [31–34]. While these methods have been useful, they only address one dimension of the COVID-19 pandemic. And because they are based on comparisons with previous periods, they become more problematical as the pandemic progresses.

As discussed throughout this article, however, the presence, magnitude, and impact of COVID-19 data limitations can be difficult to determine, especially given the newness of COVID-19, the global reach of the virus, the rapid rate of transmission (especially with the Delta variant) and the various systems attempting to capture information about it, and the evolving nature of an ongoing pandemic. It also seems likely that, as with HIV, the regions of the world experiencing the greatest impact of COVID-19 are those with the weakest data. However, longstanding initiatives, such as the INDEPTH-iShare2 network [35] have set data quality standards for its member States in low- and middle-income countries that meet or exceed those in any other data repository. According to email correspondence with Dr. Osman Sankoh, Ph.D. (oasankoh@gmail.com) on August 26, 2021, countries such as Sierra Leone have had contact tracing and surveillance systems in place well before the onset of the COVID-19 pandemic due to their experience in managing the 2014 Ebola epidemic. When fully implemented, these will likely improve data quality for COVID-19 impact studies.

Researchers must think critically about potential problems with the COVID-19 data sources over the specific time periods and particular locations they have chosen to analyze. Furthermore, to address these problems, we recommend researchers choose an appropriate design (and the data it requires). Haber and colleagues [4, 5] identify designs that are most appropriate for NPI impact analysis. In this piece, we specifically focus on designs that are robust to common data problems. We additionally recommend researchers conduct checks or sensitivity analyses of the results to data sources and design choices. Regardless of the approaches taken, researchers should be explicit about the kind of data problem or other biases that the design choices and sensitivity analysis is addressing. On-line supplements are a good place to provide the results of these analyses in sufficient detail for readers to assess their credibility.

## Data Availability

Data sharing is not applicable to this article as no datasets were generated or analyzed during the current study.

## Abbreviations

CDC: U.S. Centers for Disease Control and Prevention
COVID-19: Coronavirus disease 2019 (the illness caused by the SARS-CoV-2 virus)
ECDC: European Centre for Disease Prevention and Control
INDEPTH: International Network for the Demographic Evaluation of Populations and Their Health
ITS: Interrupted time-series analysis
NPIs: Non-pharmaceutical interventions
